# The Impact of Bariatric Surgery on the Muscle Mass in Patients with Obesity: 2-Year Follow-up

**DOI:** 10.1007/s11695-021-05815-x

**Published:** 2021-11-30

**Authors:** Marta Comas Martínez, Enzamaria Fidilio Meli, Fiorella Palmas Candia, Francesca Filippi, Ramon Vilallonga, Efrain Cordero, Irene Hernández, Alba Zabalegui Eguinoa, Rosa Burgos, Anna Vila, Rafael Simó, Andreea Ciudin

**Affiliations:** 1 Endocrinology and Nutrition Department, Hospital Universitari Vall d’Hebron, Diabetes and Metabolism Research Unit, Vall d’Hebron Institut de Recerca (VHIR), Universitat Autònoma de Barcelona, Pg Vall d’Hebron 119-129, 08035 Barcelona, Spain; 2 Research Group M3O, Methodology, Methods, Models and Outcomes of Health and Social Sciences, Faculty of Health Sciences and Welfare, University of Vic - Central University of Catalonia, Barcelona, Spain; 3 Clinical Pharmacology Department, University Hospital Vall d’Hebron, Barcelona, Spain; 4 Endocrine, Metabolic and Bariatric Unit, General Surgery Department, Hospital Universitari Vall d’Hebron, Universitat Autònoma de Barcelona, Barcelona, Spain; 5 Centro de Investigación Biomédica en Red de Diabetes y Enfermedades Metabólicas Asociadas (CIBERDEM), Instituto de Salud Carlos III (ISCIII), Madrid, Spain

**Keywords:** Morbid obesity, Fat-free mass, Bariatric surgery, Sarcopenic obesity

## Abstract

**Purpose:**

Bariatric surgery (BS) induces a significant and sustained weight loss in patients with severe obesity (SO). Nevertheless, apart from significantly reducing body fat, fat-free mass (FFM) might also be lost. At present, there is little and controversial data in the literature regarding the impact of BS on FFM. In recent years, bioimpedance (BIA) has emerged as a reliable test to assess body composition easily to use in the daily clinical practice. On the bases, the aim of the present study is to evaluate the impact of BS on the FFM, evaluated by means of BIA.

**Material and Methods:**

This is a prospective, observational study, including consecutive patients with SO that underwent BS between February 2018 and February 2019 at our center. At baseline, 1, 6, 12, and 24 months after the BS, all the patients underwent complete medical history, physical and anthropometric evaluation, and body composition assessment by means of BIA (using Bodystat QuadScan4000®).

**Results:**

Eighty-five patients with SO were recruited, 72.9% females, aged 45.54 ± 9.98 years, pre-BS BMI 43.87 ± 6.52 kg/m^2^. FFM significantly decreased continuously after BS at all timepoints. The loss of FFM 24 months post-BS accounted for approximately 21.71 ± 13.9% of the total weight loss, and was independent of BS technique or protein metabolism. Pre-BS HOMA-IR and FFM were independent predictors of FFM at 24 months.

**Conclusions:**

Significant and early loss of FFM in patients with SO that undergo BS was seen, not related to protein metabolism parameters or the BS technique used, suggesting an independent mechanism.

**Graphical abstract:**

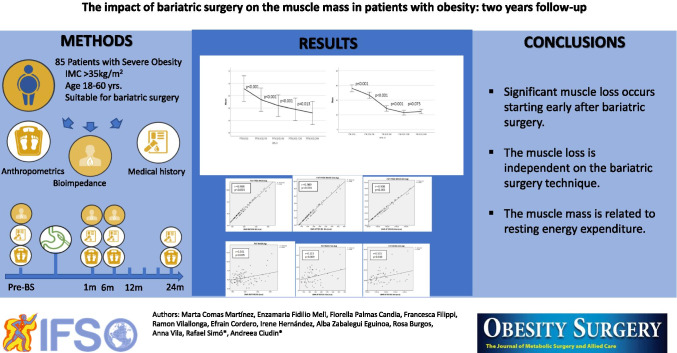

## Introduction

The prevalence of obesity has increased worldwide over the last 50 years, reaching pandemic levels, in particular severe obesity (SO). Obesity represents a significant public health challenge, and it is associated with a significant economic burden on the health systems of developed countries, mainly due to the associated comorbidities, in particular type 2 diabetes (T2D) [1–3]. Additionally, the loss of muscle mass and/or function, also known as sarcopenia, was related to metabolic disorders, like T2D, aging, and poor quality of life. [4, 5]. Sarcopenic obesity (OS) is the combination of low muscle mass and strength with increased fat mass, and it has been associated with adverse health outcomes [6]. A recent meta-analysis found that the presence of OS was associated with a higher risk (OR 1.38, 95% CI [1.27–1.50]) of T2D than with each condition separately (obesity or sarcopenia) [7]. The complete underlying mechanism is still unclear, but it seems that there is a bi-directional relationship, having as main factors chronic inflammation and insulin resistance (IR).

Physiologically, the maximum level of skeletal muscle mass and strength is reached between 30 and 50 years of age, and starting from this point, the muscle mass is decreasing as part of the aging process [8]*.* Nevertheless, several conditions can alter the physiological evolution of the muscle mass at earlier ages, such as associated obesity, rapid weight loss after diet, and physical inactivity.

The use of rapid weight-loss diets, such as very-low-calorie diet (VLCD), has been shown to have a significant weight loss effect and a significant impact on muscle mass reduction. The impact on muscle mass has been seen to be greater than the impact on fat mass [9].

Different methods are available to assess muscle mass and body composition such as bioimpedance analysis (BIA), dual-energy X-ray absorptiometry (DXA), computed tomography (CT), and magnetic resonance imaging (MRI). DXA is still considered the *gold standard* method in clinical practice and investigation—and used the diagnosis criteria EWGSOP2 for sarcopenia [10]. However, DXA is expensive, needs particular space, has subject-related limitations (maximum weight 160 kg), and provides only quantitative skeletal muscle mass evaluation. On the other hand, BIA is a relatively simple, quick, and non-expensive method for assessing body composition [11–13] and provides data on the muscle quantity and estimated quality.

Bariatric surgery (BS) is at present the most successful treatment for SO, in terms of significant and sustainable weight loss, leading to an important improvement in obesity-related comorbidities and quality of life [14]. At present, there is little and controversial data regarding the relationship between bariatric surgery and skeletal muscle mass. Furthermore, the data and the methods that were used in the different studies are heterogeneous (some used muscle mass estimation based on mathematical equations, while others use more direct methods) [15, 16].

On these bases, the aims of the present study were (a) to evaluate the impact of BS on FFM in patients with MO and (b) to explore biomarkers for FFM evolution after the BS.

## Material and Methods

We performed a prospective, observational study, including patients with SO, consecutively attended at the Morbid Obesity Unit of our centre, that underwent BS between February 2018 and February 2019. The present study, part of the PREDIBAR trial (NCT 03784508), was approved by the local Ethics Committee (PR(AG)320/2018) and carried out in accordance with the Declaration of Helsinki. All the patients signed the informed consent form before the inclusion in the study.

Inclusion criteria are as follows: (a) age between 18 and 60 years, (b) SO fulfilling criteria for BS according to our protocol (BMI > 40 kg/m^2^ regardless of the comorbidities or BMI > 35 kg/m^2^ with at least 1 comorbidity related to obesity, (c) previous accomplishment of the preoperative protocol for BS at our site, (d) written informed consent form.

Exclusion criteria are as follows: (a) patients undergoing evaluation for a second-step surgery; (b) unable to perform the post-BS follow-up at our site at least during 2 years; (c) subjects unable perform BIA (e.g. limb amputation, unwilling, and unable to fast for more than 8 hours); (d) presence of other conditions that can affect the muscle mass as per investigator criteria (e.g. immobilization, myopathies, and endocrinopathy such as Cushing disease); (e) severe concomitant pathology (cardiovascular, cerebrovascular, pulmonary, renal, or neoplastic) that can limit the participation in the study as per investigator criteria; (f) the use of drugs that can affect the muscle mass (e.g. corticosteroids); (g) active abuse of drugs or alcohol; (h) uncontrolled psychiatric illness or eating disorders.

All patients underwent baseline, at 1, 6, 12, and 24 months after the BS performance: complete medical history, physical, anthropometric, as per preoperative protocol for BS at our site. Additionally, biochemical analysis was performed including HbA1c, insulin levels, homeostatic model assessment for insulin resistance (HOMA-IR), and sensitive parameters from protein metabolism (transthyretin).

- HOMA-IR was calculated using the formula: [Fasting glucose (mmoL/L) * fasting insulin (μUI/mL)]/22.5
- BMI was calculated by the formula: weight (kg)/height (m^2^)
- %Excess of weight loss (%EWL) was calculated by the formula: (W (kg) initial − W (kg) final)/(W (kg) initial − W (kg) ideal) * 100
- %Total weight loss (%TWL) was calculated by the formula: (W (kg) initial − W (kg) final)/(W (kg) initial) * 100

Body muscle mass was evaluated by means of BIA [17]. The BIA device used in our study was Bodystat QuadScan4000®, a multi-frequency device. The measurement is performed by placing two electrodes on the wrist and hand and two electrodes on the foot and ankle on the same side of the body. BIA can be carried out both in the outpatient clinic and in the hospitalization floors since these devices can be transported easily [18]. The patients included in the study were asked to fulfil the standardized conditions to perform a BIA: avoid physical exercise the previous 8 h; fasting 6–8 h before the measurement, including water; have previously removed the hair from the limbs and all metal objects that may interfere with the measurement; if the patient wears a prosthesis or implant, the measurement is made on the opposite side [19].

The variables that were collected from the BIA evaluation were fat mass (FM) (kg), fat-free mass (FFM) (kg), fat-free mass index (FFMI) (kg/m^2^), and phase angle (PA) (°). The PA is considered an indicator of cellular integrity; it allows the interpretation of the capacity of the muscle cell to transmit the electrical stimulus produced by the BIA apparatus. Low PA values have been correlated with a worse prognosis and higher comorbidities [17] [20].

### Statistical Analysis

BM SPSS statistical software version 24 was used. Continuous variables are expressed as means ± standard deviation (SD) for normal distributed variables and median ± interquartile range (IQR) for non-normal distributed variables. Categorical variables are expressed with percentages. For differences between groups in continuous variables ANOVA, Student’s *t* test or Mann-Whitney *U* test was used while *χ*^2^ was used for categorical variables. Spearman correlation and regression logistics analysis were used to explore the relation between different variables. A *p*-value < 0.05 was considered statistically significant.

## Results

A total of 90 consecutive patients with SO undergoing preoperative evaluation for BS at our site were recruited. Eighty-five patients underwent BS and had at least 24 months of follow-up at our site and were included in the study. The baseline characteristics of the patients are shown in Table 1.

**Table 1:** The baseline characteristics of the patients included in the study

*N*	85
Gender (females %)	62 (72.9%)
Age (years) mean ± SD	45.54 ± 3.38
Pre-BS BMI (kg/m^2^) mean ± SD	43.87 ± 6.52
Roux-en-Y gastric bypass (RYGB) (%)	48 (57%)
Sleeve gastrectomy (SG) (%)	36 (43%)
HbA1c (% DCCT) mean ± SD	5.89 ± 0.96
HOMA-IR mean ± SD	6.13 ± 4.26
Type 2 diabetes (%)	40 (47%)
Arterial hypertension (%)	37 (43.5%)
Hypercholesterolemia (%)	22 (25.9%)
Sleep apnea (%)	50 (58.8%)

*BMI*, body mass index; *BS*, bariatric surgery; *HOMA-IR*, homeostatic model assessment for insulin resistance; *HbA1c (% DCCT)*, glycated haemoglobin (% Diabetes Control and Complications Trial)

Patients with T2D were treated with diet (100%), metformin (90%), aGLP-1 (62.4%), iSGLT-2 (13.2%), iDPP4 (45.1%), and insulin (2.3%). For the calculation of HOMA-IR, the patients treated with insulin were excluded. The evolution of weight, body composition evaluated by BIA and biochemical analysis after BS, is shown in Table 2. All the patients presented remission of T2D, according to ADA guidelines [21] after the BS and was maintained at 24 months.

**Table 2:** Evolution after bariatric surgery

Parameters	Baseline			1 month after BS			6 months after BS			12 months after BS			24 months after BS		
	Global	RYGB	SG	Global	RYGB	SG	Global	RYGB	SG	Global	RYGB	SG	Global	RYGB	SG
Fat-free mass (kg)‡	66.47 ± 14.49	65.68 ± 4.09	66.61 ± 15.46	62.03 ± 12.84 ^a^	62.12 ± 11.96	61.91 ± 14.11	58.94 ± 11.89^a,b^	59.12 ± 11.18	58.68 ± 12.99	56.87 ± 12.60^a,b,c^	56.37 ± 2.02	57.53 ± 13.53	55.48 ± 13.97^a,b,c,e^	54.34 ± 15.04	55.66 ± 12.91
Fat mass (kg)‡	54.97 ± 15.86	52.84 ± 11.32	57.21 ± 19.92	46.42 ± 13.89^a^	44.64 ± 11.42	48.79 ± 16.52	28.60 ± 12.68^a,b^	26.27 ± 10.31	31.89 ± 14.97	23.68 ± 11.99^a,b,c^	21.44 ± 9.77	26.64 ± 14.06	25.43 ± 8.89^a,b,c^	24.39 ± 7.83	27.78 ± 0.23
Phase angle (°)‡	6.79 ± 0.86	6.79 ± 0.91	6.96 ± 0.74	6.26 ± 0.86^a^	6.08 ± 0.84	6.57 ± 0.82^h^	5.54 ± 0.78^a,b^	5.39 ± 0.87	5.76 ± 0.59^h^	5.68 ± 1.10^a^	5.438 ± 1.04^h^	5.86 ± 0.68^h^	5.78 ± 0.96^a^	5.63 ± 0.96	6.00 ± 0.94^h^
Basal metabolism rate (kcal)‡	1960.51 ± 378.49	1928.49 ± 366.01	1984.25 ± 402.88	1838.84 ± 361.50^a^	1838.4 ± 309.61	1799.6 ± 425.15	1777.33 ± 303.71^a^	1767.83 ± 282.93	1790.74 ± 334.79	1726.68 ± 318.84^a,b,c^	1707.05 ± 302.09	1752.61 ± 343.61	1722.86 ± 349.15^a,b,c^	1712.05 ± 75.20	1727.06 ± 323.26
Fat-free mass loss from TWL (%)‡	NA	NA	NA	30.88 ± 20.74	29.56 ± 18.95	32.64 ± 23.06	26.46 ± 8.76^b^	26.37 ± 10.06	26.58 ± 6.79	19.47 ± 12.63^b,c^	19.98 ± 10.30	20.09 ± 15.19	21.71 ± 13.90^c,e,f^	23.20 ± 9.96	20.94 ± 17.60
BMI (kg/m^2^)‡	43.82 ± 6.35	42.47 ± 5.06^h^	45.85 ± 7.78^h^	38.43 ± 8.48^a^	37.71 ± 4.84	40.45 ± 9.69	31.81 ± 5.92^a,b^	30.38 ± 4.43^h^	33.81 ± 7.14^h^	29.47 ± 6.16^a,b,c^	27.89 ± 4.42^h^	31.57 ± 7.48^h^	28.14 ± 8.51^a,b^	25.75 ± 9.16^h^	31.59 ± 6.26^h^
%EWL‡	NA	NA	NA	24.25 ± 14.86	26.85 ± 11.58	20.78 ± 17.94	65.61 ± 22.03^b^	69.59 ± 21.79^h^	60.15 ± 21.48^h^	79.80 ± 24.30^b,c^	82.07 ± 25.14	75.31 ± 26.49	78.67 ± 26.98^b,c,d^	82.29 ± 24.04^h^	73.77 ± 30.59
%TWL‡	NA	NA	NA	10.15 ± 4.95	10.86 ± 3.46	9.20 ± 6.37	27.30 ± 6.34^b^	28.46 ± 5.89^h^	25.7 ± 6.66^h^	33.84 ± 7.43^b,c^	33.84 ± 7.46	31.58 ± 7.88	33.50 ± 9.57^b,c,d^	34.39 ± 8.50^h^	32.30 ± 11.01
HbA1c (% DCCT)	5.97 ± 1.10	6.01 ± 1.17	5.72 ± 0.53	5.69 ± 0.89^a^	5.78 ± 1.09	5.57 ± 0.52	5.26 ± 0.47^a,b^	5.26 ± 0.51	5.26 ± 0.43	5.32 ± 0.47^a,b^	5.29 ± 0.48	5.32 ± 0.45	5.42 ± 0.64^a,b^	5.40 ± 0.69	5.44 ± 0.57
HOMA-IR	6.31 ± 4.00	5.63 ± 3.34	6.85 ± 5.28	1.43 ± 0.48^a^	1.45 ± 0.43	2.25 ± 0.92	1.68 ± 1.31^a,b^	1.31 ± 0.64^h^	2.13 ± 1.71^h^	1.59 ± 1.15^a,c^	1.16 ± 0.53^g^	2.32 ± 1.83^g^	1.69 ± 1.34^a,d^	1.45 ± 1.34	1.92 ± 1.33
Transthyretin (mg/dl) (20–40)	23.65 ± 4.60	23.32 ± 4.92	23.97 ± 4.44	18.89 ± 4.58^a^	18.89 ± 4.58^h^	20.63 ± 3.13^h^	19.86 ± 3.86^a^	18.54 ± 4.13^g^	21.36 ± 2.93^g^	22.56 ± 3.64^a,b,c^	21.59 ± 3.86	23.63 ± 3.12	25.24 ± 4.66^a,b,c,d^	24.24 ± 5.07	26.47 ± 3.89
Albumin (g/dl) (3.5–5.2)	4.14 ± 0.51	4.20 ± 0.21	4.16 ± 0.26	4.22 ± 0.30	4.19 ± 0.22	4.25 ± 0.38	4.08 ± 0.28^b^	4.06 ± 0.26	4.10 ± 0.29	4.06 ± 0.46	4.03 ± 0.60	4.09 ± 0.24	5.22 ± 5.99	6.07 ± 8.01	4.17 ± 0.28
FFMI (kg/ m^2^)‡	23.79 ± 3.2	23.19 ± 2.74	24.61 ± 3.64	22.30 ± 2.85^a^	21.83 ± 2.39	22.93 ± 3.31	21.30 ± 2.63^a,b^	20.88 ± 2.22	21.90 ± 3.05	20.75 ± 2.98^a,b,c^	20.12 ± 2.55	21.57 ± 3.33	20.20±3.33^a,b,c,d^	19.54 ± 2.71	21.04 ± 3.87

*BS*, bariatric surgery; *BMI*, body mass index; *EWL*, excess of weight loss; *TWL*, total weight loss; *HOMA-IR*, homeostatic model assessment for insulin resistance; *HbA1c*, glycated haemoglobin; *FFMI*, fat-free mass index

^a^Significantly different with respect to baseline at *p* < 0.001

^b^Significantly different with respect to 1 month at *p* < 0.001

^c^Significantly different with respect to 6 months at *p* < 0.001

^d^Significantly different with respect to 12 months at *p* < 0.001

^e^Significantly different with respect to 12 months at *p* < 0.05

^f^Significantly different with respect to 1 month at *p* < 0.05

^g^Significantly different between bariatric surgery techniques at *p* < 0.001

^h^Significantly different between bariatric surgery techniques at *p* < 0.05

‡Repeated measures ANOVA, *p* < 0.05

**Fig. 1:**
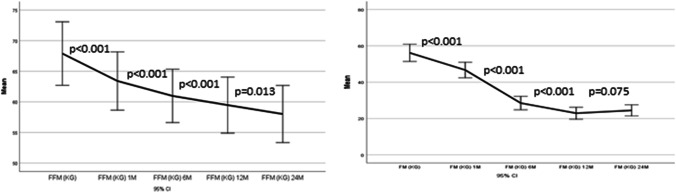
Evolution of the FFM and FM during 24-month follow-up. FFM fat-free mass, FM fat mass, M months—reviewer 2

The FFM significantly decreased 1 month after the BS and continued to drop at 24 months. It should be noted that starting from 1 month until the end of the follow-up, FFM loss represented > 20% of the TWL (Table 2). FM also significantly decreased starting from 1 until 12 months after the BS. Between months 12 and 24 after the BS, FM stabilized and presented a slight tendency to increase, although without reaching statistical significance, as reflected by Fig. 1.

**Fig. 2:**
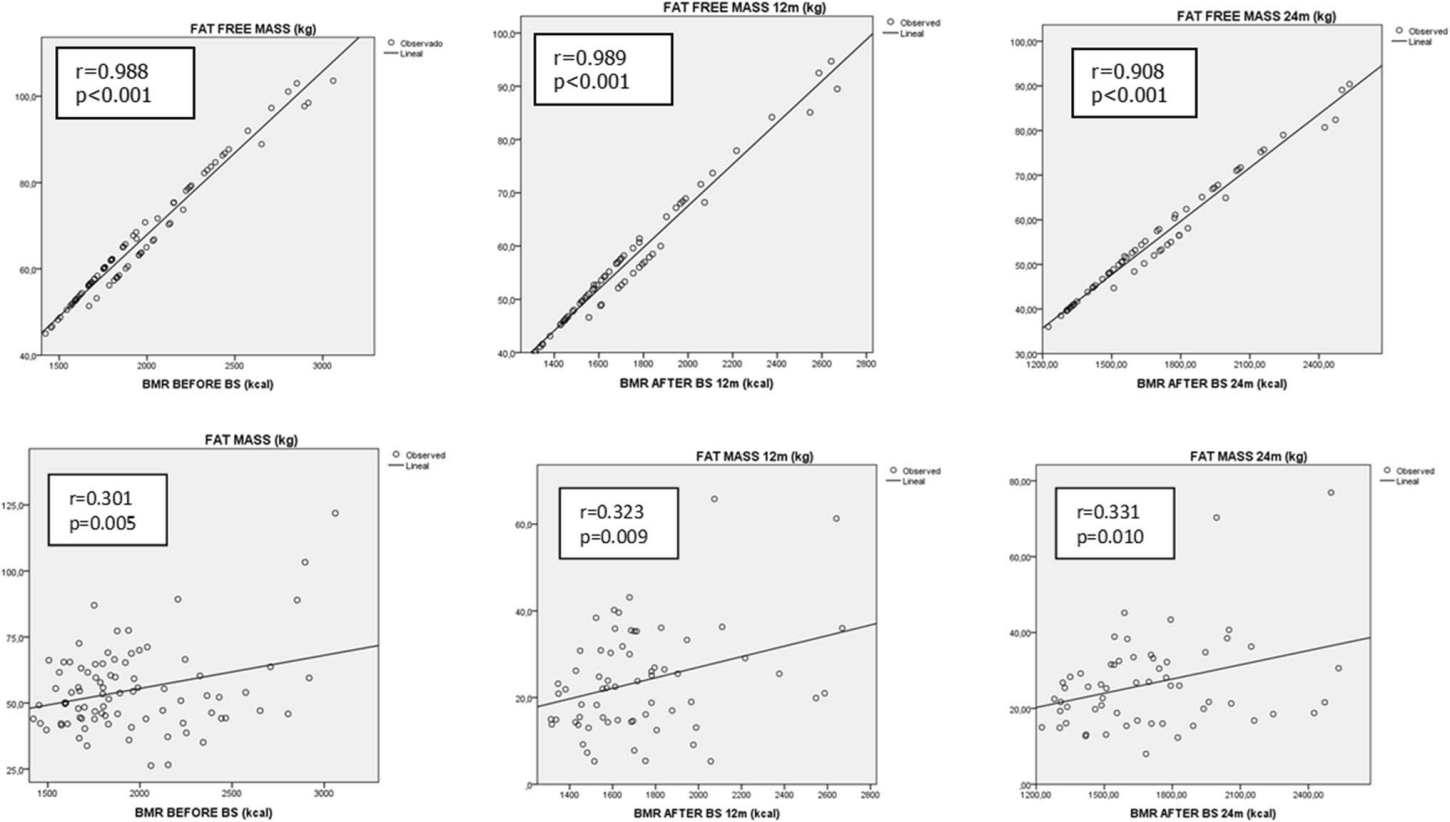
Correlation between basal metabolism rate and body composition. BMR basal metabolism rate, BS bariatric surgery

Additionally, basal metabolism rate (BMR), measured by BIA, significantly decreased 1 month after the BS compared to baseline and continued to decrease during the follow-up (Table 2). A positive correlation was found between FFM and the BMR at all timepoints, respectively. By contrast, FM showed no relation with the BMR as reflected by Fig. 2.

Regarding the two BS techniques that were used in the study (RYGB and SG), as expected, RYGB was associated with greater global EWL and TWL than SG. No significant differences were seen between the two techniques in terms of body composition and biochemical analysis (HOMA-IR and transthyretin), except for the PA that was significantly lower after the RYGB, as reflected by Table 2. We found significantly lower levels of PA after the BS in all patients, when compared to data in the literature from subjects with normal weight, of similar age and gender [22] (Table 3).

**Table 3:** The BS impact on the Phase angle levels in all patients

	Baseline	1 month	6 month	12 months	24 months	Normal values [22]	*p*‡
**Females**							
18–20 years	7.86 ± 1.06	6.46 ± 0.3^a^	6.25 ± 0.59^a,b^	6.15 ± 0.6^a,b,c^	6.13 ± 0.38^a,b,c,d^	7.04 ± 0.85	0.001
21–29 years	7.4 ± 0.74	6.14 ± 0.85^a^	5.80 ± 0.55^a,b^	5.9 ± 0.76^a,b,c^	5.68 ± 0.53^a,b,c,d^	6.98 ± 0.92	0.0021
30–39 years	6.6 ± 0.59	5.96 ± 0.76^a^	5.39 ± 0.66^a,b^	5.15 ± 0.77^a,b,c^	5.07 ± 0.80^a,b,c,d^	6.91 ± 0.85	< 0.001
40–49 years	6.12 ± 0.9	5.7 ± 0.12^a^	4.89 ± 0.85^a,b^	4.85 ± 1.04^a,b,c^	5.07 ± 0.16^a,b,c,d^	6.87 ± 0.84	< 0.001
**Males**							
18–20 years	NA	NA	NA	NA	NA	7.9 ± 0.47	NA
21–29 years	7.7 ± 0.3	7.3 ± 1.12^a^	7.05 ± 0.3^a,b^	7.15 ± 0.4^a,b,c^	6.35 ± 0.91^a,b,c,d^	8.02 ± 0.75	< 0.001
30–39 years	7 ± 0.56	6.7 ± 0.01^a^	6.45 ± 1.47^a,b^	6.25 ± 0.75^a,b,c^	6.14 ± 0.54^a,b,c,d^	8.01 ± 0.85	< 0.001
40–49 years	6.75 ± 0.79	6.85 ± 0.46^a^	5.63 ± 0.68^a,b^	5.58 ± 0.61^a,b,c^	5.73 ± 0.70^a,b,c,d^	7.76 ± 0.85	< 0.001

^a^Significantly different respect to baseline at *p* < 0.001; ^b^significantly different with respect to 1 month at *p* < 0.001; ^c^significantly different with respect to 6 months at *p* < 0.001; ^d^significantly different with respect to 12 months at *p* < 0.001; ‡repeated measures ANOVA, *p* < 0.05

A multiple regression analysis, including variables as age, HbA1c levels, baseline FFM, baseline FM, baseline transthyretin, and HOMA-IR, was performed. We found that HOMA-IR and baseline FFM were the only independent predictors of FFM at 24 months after the BS as reflected by Table 4.

**Table 4:** Independent parameters related to the FFM at 24 months after the BS

Coefficients^a^									
Model	Unstandardized coefficients		Standardized coefficients	*t*	Sig.	95.0% confidence interval for B		Collinearity statistics	
	*B*	Std. error	Beta			Lower bound	Upper bound	Tolerance	VIF
1 (constant)	− 8.750	8.300		− 1.054	.300	− 25.725	8.225		
Age	.009	.076	.006	.116	.908	− .147	.164	.884	1.131
FFM (kg)	.843	.047	.933	17.966	.000	.747	.939	.813	1.230
Transthyretin before	− .024	.145	− .008	− .162	.872	− .320	.273	.812	1.231
HOMA-IR	.409	.192	.102	2.132	.042	.017	.802	.966	1.036
HbA1c levels	.800	.610	.065	1.313	.200	− .447	2.048	.891	1.122
FM (kg)	.041	.056	.040	.722	.476	− .075	.729	.729	1.371

^a^Dependent variable FFM (kg) 24 months

*FFM* fat-free mass, *FM* fat mass, *M* months

## Discussion

We showed in the present study that BS induces a significant and early loss of FFM after the BS, independent of protein metabolism, without significant differences between the BS techniques (RYGB and SG). In our study, BMI stabilized after 12 months post-BS. In exchange, body composition showed different profiles between 12 and 24 months: FFM continued to significantly drop while FM presented a tendency to increase, confirming the actual data in the literature [23–25] regarding the lack of precision of BMI to properly monitor the evolution after the BS.

Recently, our group showed that BS induces a significant reduction in basal metabolism rate starting 1 month after the BS in patients with extreme obesity that was associated with weight regain at 5-year follow-up [26]. It should be noted that in the study performed by Fidilio et al. [26], the BMR was measured by means of indirect calorimetry in patients with extreme obesity (BMI > 50 kg/m^2^) and study of body composition was not performed. In the present study, we found also a significant and early reduction in BMR starting 1 month after the BS, although measured by BIA that was significantly correlated with FFM at all timepoints and showed no relation with the FM. These findings suggest the role of FFM in the weight homeostasis and metabolism and point out to the importance of centring the attention on the FFM and a more personalized approach of patients with obesity that are proposed for weight loss interventions. Nevertheless, at present, there is no data in large cohorts of patients on the role of the FFM in the evolution after weight loss interventions, since body composition is not usually taken into consideration in the daily clinical practice. This fact represents an important gap in the management of the patients with obesity that urgently needs to be filled.

Additionally, there is very scarce data regarding the impact of BS on the FFM evolution. Vaurs et al. [16] identified two phenotypes of FFM evolution after BS and defined as “muscle spare” = FFM < 15% of TWL and “muscle loss” = FFM > 15% of TWL. In our study, we found reduction in FFT > 20% of TWL at all timepoints, reaching 25% after 24 months, independently of the BS technique (RYGB or SG) which according to present published data is considered significant muscle loss.

Besides FFM and FM parameters that are usually evaluated in body composition studies, recently, a new parameter has emerged as a more sensitive biomarker of muscle mass, in particular quality: the phase angle (PA) [27]. PA makes possible to interpret the muscle cell’s capacity to transmit the electrical current produced by the BIA device according to the cell’s quality. PA is an attractive index, because it is independent of body weight and was associated with poor health outcomes, quality of life, and mortality [28]. It should be noted that at present, there is very few data in the literature regarding PA normal cut-offs. Most of the studies were performed including patients in a critical state in the intensive care units or with cancer, showing levels around 4–5° [28, 29]. In a study of 1967 healthy subjects from the USA, the PA of age-matched individuals was 6.96° ± 1.10° for males and 5.97° ± 0.83° for females [30]. A larger study published by Bosy-Westphal et al. [22], including data from 210,000 healthy German individuals, proposed the cut-off for PA of 6.01° ± 0.75° for males and 5.59° ± 0.72° for females, with tenth percentile values of 5.14 and 4.79, respectively. A value below this cut-off is considered sarcopenia and low muscle mass and also quality. It should be noted that none of these studies included patients with obesity or overweight.

At present, there is no data regarding the PA in subjects with obesity and/or the impact of BS on the muscle quality evaluated by this parameter. As reflected by Table 3 from the results, in our study, we found PA levels at baseline comparable to those reported in the German study [22], although slightly lower. Nevertheless, our study was not designed to evaluate sarcopenia pre-BS and we cannot make any hypothesis on the pre-BS FFM status of our patients. Actually, at present, this point represents another important gap in the management of the patients with obesity, since there is no reliable data regarding the prevalence of sarcopenia in these subjects, especially the younger ones that are usually candidates for BS. Application of different criteria to identify sarcopenia associated to obesity may therefore currently lead to substantially and clinically unacceptable variable prevalence levels (ranged from 8 to over 50%) due the heterogeneity in the methods that were used [6]. In order to fill this gap further, large case control studies are needed in order to dispose of reliable data. In exchange, the PA was significantly lower after the BS at all timepoints compared to baseline and significantly below the normal cut-off for both females and males proposed by Bosy-Westphal et al. [22], suggesting sarcopenia. Furthermore, PA was significantly lower in the patients that underwent BYGR, despite similar FFM with SG, suggesting that RYBG might have a greater impact on the muscle quality after the BS.

As reflected by the logistic regression analysis, the important loss of FFM after the BS was independent of the age, gender, protein metabolism, or BS techniques. The only parameters associated with the FFM at 24 months after the BS in our study were pre-BS FFM and pre-BS HOMA-IR, suggesting that pre-BS conditions have an important influence on the evolution after the BS. Previous data in the literature showed that insulin resistance (IR) has an indirect relationship with the muscle mass in humans [31]. From a mechanistic point of view, interestingly, in murine models, Ostler et al. [32] demonstrated that IR, in a context of leptin signalling impairment and inflammation (that are also present in humans with obesity), induces a significant decrease in muscle size and quality. Additionally, another study in murine models performed by Wang et al. [33] showed that IR causes muscle protein degradation. Furthermore, in the same study, treatment with rosiglitazone (a drug that decreases IR) induced only a partial recovery of the muscle mass and the authors hypothesized that maybe because it did not interfere with protein synthesis.

These previous data in murine models explain our findings in the present study. Pre-BS IR might have altered the muscle mass quantity and quality and the impact of significant rapid metabolic and body weight changes induced by the BS have led to a significant and continued muscle mass loss. Furthermore, as seen in the murine model [33], the muscle mass was not recovered after the normalization of the IR. We observed the same evolution in our study, where the FFM continued to drop out at 24 months even if the HOMA-IR normalized after the first month and remained within normal limits for Spanish population [34] during the follow-up. Our findings suggest that the mechanisms of significant muscle mass loss after BS seem independent on the BS techniques or protein metabolism parameters, like transthyretin, but are far from being elucidated. This is the first study that shows a significant FFM loss after BS maintained after 24 months, having independent predictors pre-BS FFM and IR. The main limitations of our study are the lack of a control group of subjects with normal weight and the sample size. Nevertheless, the patients represented their own control and the study was aimed to evaluate the evolution during follow-up. For this reason, we consider that the main limitations had no significant impact on the results.

These findings point to the urgent need to take into consideration changing the actual clinical guidelines for the management of patients with obesity, especially those that are candidate for BS, by incorporating body composition studies (both quantitative and qualitative) in the routinary preoperatory and follow-up evaluations. BIA is a rapid, reliable, repeatable, and non-expensive test that can be easily implemented in the daily clinical practice, as part of the evaluation of patients with obesity. This action will allow to implement more personalized approach, and design-specific physical exercises, diet, and pharmacological therapy aimed at improving the pre-BS muscle mass and insulin resistance.

## Conclusions

There is a significant and early loss of FFM in patients with SO who undergo BS, which is not related to the parameters of protein metabolism or the surgical technique used, suggesting the existence of an independent mechanism.
